# Earliest “Domestic” Cats in China Identified as Leopard Cat (*Prionailurus bengalensis*)

**DOI:** 10.1371/journal.pone.0147295

**Published:** 2016-01-22

**Authors:** Jean-Denis Vigne, Allowen Evin, Thomas Cucchi, Lingling Dai, Chong Yu, Songmei Hu, Nicolas Soulages, Weilin Wang, Zhouyong Sun, Jiangtao Gao, Keith Dobney, Jing Yuan

**Affiliations:** 1 Unité Mixte de Recherche (UMR) 7209, Archéozoologie, Archéobotanique: Sociétés, Pratiques et Environnements, Centre National de la Recherche Scientifique, Muséum National d’Histoire Naturelle, Sorbonne Universités, CP 56, Paris, France; 2 Department of Archaeology, University of Aberdeen, St Mary's, Aberdeen, United Kingdom; 3 University of Chinese Academy of Sciences, Beijing, China; 4 Shaanxi Provincial Institute of Archaeology, Xi’an, China; 5 Institute of Archaeology, Chinese Academy of Social Science, Beijing, China; Chinese Academy of Sciences, CHINA

## Abstract

The ancestor of all modern domestic cats is the wildcat, *Felis silvestris lybica*, with archaeological evidence indicating it was domesticated as early as 10,000 years ago in South-West Asia. A recent study, however, claims that cat domestication also occurred in China some 5,000 years ago and involved the same wildcat ancestor (*F*. *silvestris*). The application of geometric morphometric analyses to ancient small felid bones from China dating between 5,500 to 4,900 BP, instead reveal these and other remains to be that of the leopard cat (*Prionailurus bengalensis*). These data clearly indicate that the origins of a human-cat ‘domestic’ relationship in Neolithic China began independently from South-West Asia and involved a different wild felid species altogether. The leopard cat’s ‘domestic’ status, however, appears to have been short-lived—its apparent subsequent replacement shown by the fact that today all domestic cats in China are genetically related to *F*. *silvestris*.

## Introduction

With global numbers of more than 500 million individuals, the domestic cat (*Felis catus*) is amongst the most common pet in the world today. Although modern genetic data indicates that the South-West (SW) Asian and North African subspecies of wildcat (*Felis silvestris lybica*) is the ancestor of all modern domestic cats [1], both geographic and temporal details regarding its domestication history remain largely conjecture. In Egypt, paintings dating to the 20-19^th^ century BC (Middle Kingdom, 12th Dynasty) have long been considered the earliest clear evidence for cat domestication [2,3]. However, the recent discovery of male and female cat skeletons (along with four kittens belonging to two different litters) at the Egyptian Predynastic elite cemetery of Hierakonpolis (HK6), provides new zooarchaeological evidence for the earlier cultural control of cats during the Naqada IC-IIB period (c. 5800–5600 cal BP—calibrated radiocarbon years before present) [4].

Minor morphological differences observed between the purported wild ancestor (*Felis silvestris lybica*) and early domestic cats, led scholars to suspect that cat domestication began even earlier than Ancient Egyptian times—at least prior to when visible osteological changes occurred on their skeletons and teeth. Therefore, the presence of cat remains at much earlier archaeological sites from Cyprus (dating from 10,800 to 8,000 cal BP, i.e. Late Pre-Pottery Neolithic A, [PPNA] to Khirokitia phase) [5–8] and another complete cat skeleton tightly associated with a human PPNB burial dating to 9500–9000 cal BP at Shillourokambos [6], provide intriguing evidence for their human introduction to the island and suggest that at least some cats of apparently unchanged wild morphology were not only commensal, but already on their way to being domesticated in SW Asia during the early Holocene.

This early introduction of cats to Cyprus from somewhere in continental SW Anatolia or the Levant may have been a deliberate act on the part of the PPN settlers to deal with a new problem—commensal mice (*Mus cypriacus/domesticus*). Indeed, the latter began to proliferate with the beginnings of cereal and legume cultivation on the near continent and on Cyprus [7,9–10]. Thus, the origins of our relationship with cats may have been indirectly initiated by the onset of farming in SW Asia, more than 10,800 years ago—a good example of the ‘commensal pathway’ to domestication [6,11–13].

The earliest unquestionable evidence of cats smaller in size than the wildcat (and therefore assumed domestic) dates to the Uruk period (5500–5000 cal BP) from Tell Sheikh Hassan [14]. This evidence suggests that it took several millennia for the domestication process to manifest itself in cats—a likely explanation being the low morphological variability of *F*. *silvestris* [15] and/or the extended nature of the commensal relationship with humans.

Recent discoveries have been used to claim cat domestication may also have occurred in Northwest China, following essentially the same commensal pathway as in Egypt and/or SW Asia [16]. Based on the osteometric analyses of eight felid bones found in the Middle-Late Yangshao (Middle Neolithic) agricultural settlement of Quanhucun, Shaanxi Province (directly dated to 5560–5280 cal BP), Hu et al. [16] showed that the Quanhucun cat measurements were smaller than the modern wildcat *F*. *s*. *lybica* and that they also fell within the size range of modern domestic cats. In addition, when collagen stable isotope analysis was undertaken on the same remains, the high δ^13^C and δ^15^N values from two of the three Quanhucun cat bones were interpreted as evidence for a diet based on rodents eating the waste of millet farmers (millet is characterized by high δ^13^C values because of its photosynthesis of C4 plants). In addition, a mandible with surprisingly worn teeth (along with a tibia showing a much lower δ^15^N value) were taken to indicate that at least some cats from the Quanhucun site “scavenged among or [were] fed by people” (p. 116) and that the commensal status of the Yangshao period cats from Quanhucun could have ultimately resulted in their domestication[16].

On the basis of these data, the authors claimed that the evidence from China provided “the earliest known evidence for a commensal relationship between people and cats” [16] (p. 116), even though the introduction of cats to Cyprus has been shown to be some 4,500 years earlier. Bar-Oz and collaborators have questioned the low δ^15^N value obtained by Hu et al. for one of the Quanhucun cats, arguing that this value is diagnostic of herbivores and cats are obligate carnivores [17]. They agreed that the Quanhucun cats were likely commensal, but considered that the suggested link between the current evidence and the trajectory of cat domestication in China is tenuous and that, in the absence of a definitive taxonomic identification of the small Quanhucun felid, the data can only be interpreted as evidence of commensalism, not domestication.

**Fig 1:**
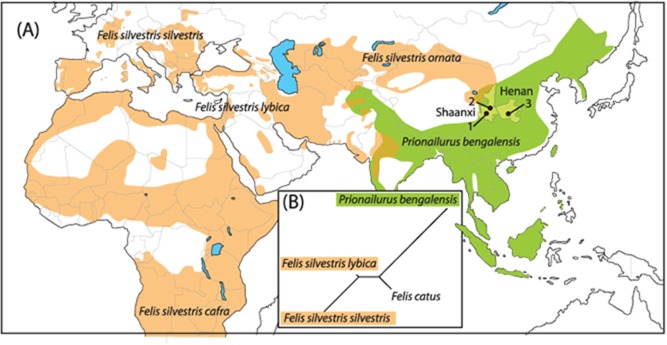
Modern distribution of wild felid species, archaeological site location and mandible shape relationship between modern wild felid species and domestic cat. **(A)**, Modern Old World distribution of the different wild cat subspecies (*Felis silvestris*) and the leopard cat (*Prionailurus bengalensis*), and location of the three Middle-Late Neolithic sites of the Shaanxi and Henan Provinces (China) analyzed in this paper: 1, Quanhucun, 2, Wuzhuangguoliang, 3, Xiawanggang (Redrawn from [http://maps.iucnredlist.org/map.html?id=60354712] and [http://maps.iucnredlist.org/map.html?id=18146] under a CC BY license, with permission from IUCN Red List of Threatened Species; S1 Text.; CAD I. Carrère); **(B)**, Phenotypic relationship (unrooted neighbour joining tree) built on mandible shape distances between modern domestic cat (*F*. *catus*), leopard cat (*P*. *bengalensis*) and the two relevant sub-species of wild cat (*F*. *s*. *silvestris; F*. *s*. *lybica*) from our analyses.

Although the past distribution of small felids in Asia remains largely unknown, the relatively small amplitude of the Late Holocene climate change suggests that it should not differ significantly from their modern ranges. Today, four small wild felids live in Shaanxi Province [1,18–22]: the Central Asian wild cat (*Felis s*. *ornata*; 1.0–2.1 kg), the Chinese Mountain cat (*Felis s*. *bieti*; 4.0–6.5 kg), the Pallas’s cat (*Otocolobus manul*; 1.8–4.0 kg) and the North Central China subspecies of the leopard cat (*Prionailurus b*. *bengalensis*; 1.5–3.8 kg). *F*. *s*. *bieti* can be excluded as a possible match for the published Quanhucun cat because it is much larger and today lives only in mountainous areas. *O*. *manul* is phylogenetically intermediate between *F*. *silvestris* and *P*. *bengalensis*, but its head and mandible are much more massive [23] and, therefore, cannot be confused with the two other species. Consequently, the Quanhucun cats must either derive from local *F*. *s*. *ornata* or *P*. *b*. *bengalensis* (Fig 1a), or alternatively represent early domestic cats (*F*. *s*. *lybica*) imported from SW Asia.

The close phenotypic proximity of these taxa [1, 23–24] prevents their definitive identification by traditional osteometric techniques from isolated and fragmented zooarchaeological remains. We, therefore, applied two dimensional landmark-based geometric approaches to five archaeological Chinese cat mandibles, which included the specimens from Quanhucun studied by Hu et al. [16], and several additional small felids recovered from two Middle and Late Neolithic sites located in Shaanxi and Henan Provinces (Fig 1a, Table 1).

**Table 1:** Archaeological information for the five Chinese cat specimens.

Site	Cultural phase	Context	Skeletal part(s)	Direct radiocarbon date		Reference
				14C age BP ± 1σ	Calibrated date 2 σ	
Quanhucun	Miaodigou, Middle-late Yangshao, 6000–5000 cal BP	H172_A: Refuse pit	Left Mandible, Right Humerus, Right Pelvis, Right Femur, Right Tibia	BA110855: 4765±30	5590–5330 cal BP	[16]
Quanhucun	Miaodigou, Middle-late Yangshao, 6000–5000 cal BP	H172_B: Refuse pit	Right Mandible	BA110855: 4765±30	5590–5330 cal BP	[16]
Wuzhuangguoliang	Miaodigou/Majiayao Yangshao-Longshan, 6000–4700 cal BP	H3: Refuse pit	Sub-complete skeleton including complete skull and mandibles	XA8399: 4422±29	5267–4871 cal BP	[25]
Xiawanggang	Late Longshan, 4500–4000 cal BP	T4, H134: Refuse pit	Left Mandible, Right Ulna, Left Tibia	No direct radiocarbon date		[26]
Xiawanggang	Late Longshan, 4500–4000 cal BP	T4, Layer 7	Right Mandible, Femur	No direct radiocarbon date		[26]

Here we report the results of the comparative study of five archaeological mandibles with modern domestic (*F*. *catus*; N = 13) and wild cats: the Northern Chinese sub-species of leopard cat (*P*. *b*. *bengalensis*; N = 10), the European subspecies of wildcat (*F*. *s*. *silvestris*; N = 29) and the SW Asian and North African subspecies of wildcat (*F*. *s*. *lybica*; N = 44) (S1 Table). In order to reaffirm the identification of the Cypriot PPNB cats as *F*. *silvestris* [6], we also included five mandibles from the site of Shillourokambos (dated to 9500–9000 cal. BP [27]) in the analyses.

### Archaeological specimens and contexts

The site of Quanhucun (Hua County, Shaanxi prov.) is an agricultural settlement dated to the Middle-Late Yangshao period [16]. Together with remains of sika deer, caprines and large bovids, eight cat bones were recovered from three different refuse pits (H172, H35, H130). Six, including the two mandibles analyzed here, were recovered from a large refuse pit (H172). A tibia from the same pit provided collagen δ^13^C and δ^15^N isotope values of -16.1‰ and 8.2‰ respectively [16]. A cat bone from the same pit (H172) has been directly dated to 5590–5330 cal BP (4765 ± 30 BP) [16]. Two hemi-mandibles (right and left) have teeth that are both very worn and thus likely derive from the same individual.

The site of Wuzhuangguoliang (Jingbian County, Shaanxi prov.) is also an agricultural settlement dated to the transition between the Yangshao and Longshan cultures. During excavations in 2001, refuse pit H3 produced pig, hare and weasel bones, together with a nearly complete felid skeleton laid on its left side (S1 and S2 Figs). The left calcaneus has been directly dated using AMS ^14^C to 5267–4871 cal BP (Table 1). Based on classical measurements (S2 Table), the specimen was initially identified as *Felis silvestris* [25]. Features of skull morphology (namely the shape of the tympanic bulla) allow us to exclude *F*. *manul* as a likely candidate. However, values for traditional cranial measurements all fall intermediate between our modern *F*. *silvestris* and *P*. *bengalensis* reference datasets.

The site of Xiawanggang (Xichuan county, Henan prov.) is a settlement occupied from the Late Yangshao culture through to the Han Dynasty [26]. Recent excavations (2008–2010) of unit T4 recovered felid bones, together with other animal remains that included pig, deer, cattle, bear, bird and fish. Cat bones derive from two different contexts, both dated to the Late Longshan culture (4500–4000 cal BP): refuse pit H134 provided a left mandible, a right ulna and a left proximal tibia; layer 7—a right mandible and a distal femur. None of these specimens have been directly radiocarbon dated, but they were from well-controlled archaeological contexts with associated datable artefacts and little evidence of stratigraphic disturbance. Even though *F*. *silvestris* is not present today in the Henan province, it is possible that it was living there during the Neolithic.

The five mandibles studied from these sites represent at least four separate individuals and span a time range of *circa* 1,500 years—from the Middle Yangshao to the Late Longshan cultures.

## Results

### Morphometric differentiation of modern felid taxa

Based on the 11 landmarks recorded on the mandibles, we established that the leopard cat and the European and the SW Asian wildcats clearly differ in shape (F(42, 264) = 3.46, *p* = 4.8e^-10^), but not in size (Chi^2^ = 6.36, df = 3, p = 0.09)—the vertical *ramus* appears to be more developed in the dorsal and posterior direction in leopard cats than in wildcats (S3 Fig). The domestic cat and the SW Asian wildcat show the closest phenotypic proximity, while the leopard and European wildcats plot at either extremes of the network (Fig 1b). Only 59.6% of specimens can be correctly identified to one of the four lineages, but 82.5% were correctly identified to the species level when only the leopard cat and the wildcats are considered.

### Identification of Chinese and Cypriot archaeological cats

**Fig 2:**
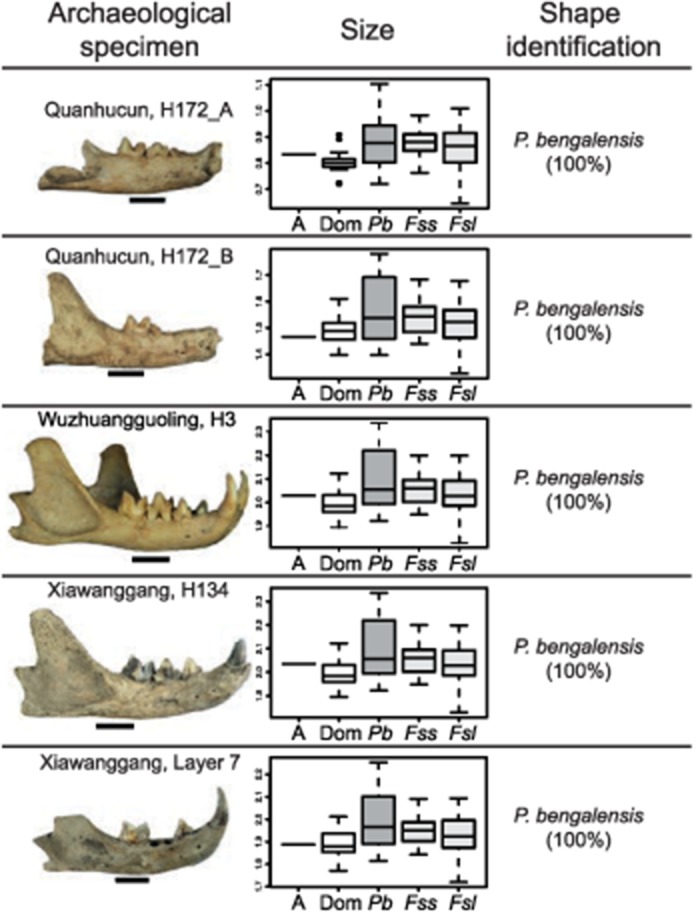
Geometric morphometric analyses of the five archaeological Chinese cat mandibles. Left column: lateral view of the mandibles—the first and the fourth specimens being transposed right side left, the scale bare represents 1cm. Middle column: Boxplot comparison of centroid size of the archaeological specimen (A), with those of modern: domestic cat (Dom), leopard cat (*Pb*), European wildcat (*Fss*) and SW Asian wildcat (*Fsl*). Right column: species identification of the specimen based on discriminant analyses computed on mandible shape variables. Percentages within brackets correspond to the probability of being identified as *Pb*.

The fragmented nature of the Chinese archaeological mandibles required the use of a subset of landmarks and the computation of separate discriminant analyses for each archaeological specimen. Nonetheless, analyses show that all Chinese archaeological felid specimens were unambiguously identified as leopard cat (*P*. *bengalensis*) (Fig 2, S3 Table). Their centroid size is relatively small, but within the range of current leopard cat specimens available in our dataset. The PPNB Cypriot cats were identified as *F*. *silvestris*, with probability ranging from 94% to 100% (S3 Table)—their size range being significantly higher than the Chinese archaeological cats (S4 Fig).

## Discussion

The mandible shape analyses demonstrate that the small felids from Middle and Late Neolithic (5,500 and 4,000 cal. BP) sites in Shaanxi and Henan Provinces display close phenotypic similarities with *P*. *bengalensis* and not with *F*. *silvestris lybica or F*. *s*. *silvestris*. We can, therefore, reject the hypothesis that these small Chinese felids are commensal or early domesticated cats introduced from SW Asia between 10,800 and 5500 cal BP, where only *F*. *s*. *lybica* have so far been identified. We cannot completely rule out the occurrence of *F*. *s*. *ornata* (the Central Asian wildcat still extant in the North of Shaanxi province) at these sites due to the lack of available samples in our study. However, previous genetic and morphometric analyses [1, 23, 24] have demonstrated that *F*. *s*. *ornata* is likely a late (divergent) relative (~Late Glacial/Early Holocene) of the other *F*. *silvestris* subspecies, whose cranial morphology is closest to *F*. *s*. *lybica* than to *F*. *s*. *silvestris*. This suggests that the morphological distances between *F*. *s*. *ornata* and the other subspecies of *F*. *silvestris* are likely smaller than that between *F*. *silvestris* and *P*. *bengalensis*. Therefore, the highly significant similarity of the five Chinese archaeological mandibles with modern *P*. *bengalensis*, strongly support their taxonomic identification as leopard cat—more precisely the north central Chinese subspecies, *P*. *b*. *bengalensis*—still extant in Shaanxi and Henan Provinces.

The leopard cat is known for its ability to adapt to human modified and cultivated environments [28–31]. Such anthropophilous behaviour makes it a good candidate for a commensal relationship with humans at the Neolithic settlements of Quanhucun, Wuzhuangguoliang and Xiawanggang, as well as other Middle and Late Neolithic settlements in China (S4 Table). As proposed for SW Asia and Egypt [3,8], grain storage and cultivation waste probably attracted rodents into early Chinese agricultural settlements, which were closely followed by leopard cats. Rodents are abundant in the Chinese Neolithic faunal record [25], but rather than the Chinese zokor (*Myospalax* sp.) (more questionably considered as commensal rodents of Chinese Neolithic villages [16]) - the most frequent anthropophilous rodents were likely Murids and more probably rats (*Rattus* sp.). Furthermore, the Myospalacinae are strictly specialised for a subterranean lifestyle [18] with no commensal behaviour observed. Their presence in archaeological contexts is, therefore, likely the result of more recent intrusive burrowing.

Our data show that the leopard cat most likely became a commensal species at North Central Chinese Neolithic sites, from at least the mid-sixth millennium cal BP, i.e. 5,000 years after the earliest evidence of commensal cats in SW Asia and their introduction to Cyprus [7,8]. However, did this commensal relationship ultimately result in its domestication? Apart from the surprisingly [17] low δ^15^N values obtained by Hu et al. [16] for one cat bone at Quanhucun (pit H130), three other lines of evidence may favour this latter hypothesis. First, the extensive wear observed on the teeth of the two mandibles from pit H172 at Quanhucun (likely the same individual) is very uncommon in small wild felids and suggests that this individual cat may have been fed by humans. Second (and in contrast with the vast majority of other broken animal bones from the sites in question), the complete articulated cat skeleton found in pit H3 at Wuzhuangguoliang is a very unusual find, similar in nature to the one found associated with a Neolithic human burial on Cyprus previously mentioned [6]. These remains are, therefore, not from simple refuse deposits but rather represent one of the rare examples of intentionality in animal burials (where the body appear to have been carefully treated and deposited in death) and, as such, implies a degree of personalisation of the individual itself [27]. Finally, all five Neolithic mandibles fall within the smallest size range of modern wild leopard cat from China (Fig 2)—echoing one of the traditionally accepted signatures of the domestication process [2,12].

Admittedly, these three lines of evidence remain somewhat tenuous and the status of the leopard cat in China during the Neolithic needs further investigation. However, we cannot exclude that at least some of the Neolithic leopard cats were already set on the domestication path through their initial commensal behaviour whilst showing little morphological change.

The leopard cat can easily be bred in captivity—even hybridizing with an American shorthair domestic cat in 1963 to produce the famous domestic Bengal breed [32]. However, the leopard cat does not appear to have contributed genetically to any extant lineages of domestic cats living in China today [1,11,15]. The domestic cat *F*. *catus* (descendant of the wildcat *F*. *s*. *lybica*) appears to have completely replaced the leopard cat from its purported commensal niche in China, at some point after the Late Neolithic. Although cat bones have been found in the Guangyangqing King burial, at Dabaotai, Beijing, dated to 45 BC (Han dynasty) [33], the earliest historical record of the presence of domestic cat (*F*. *catus*) in China dates to the Tang dynasty (AD 618–907). At that time cats were kept as pets—as well as pest (mice) controllers—in the royal palaces and local official courts. Since then, cats constantly occur in both documentary and artistic sources [34].

Were these later cats of the Han and Tang dynasties descendants of Neolithic commensal leopard cats or introduced western domestic cats? Only further archaeozoological discoveries and analyses of cat remains can answer these questions. Either way, our data has provided wholly new evidence for another possible history of cat domestication, not only by revealing a possible independent process in China, but also by adding a new (hitherto unknown) species to the pantheon of commensal/domestic animals who began their relationships with humans at the onset and spread of agriculture during the Holocene.

## Methods Summary

### Ethic statement

The Chinese specimens that we study are all curate in a public state institution in China, under the responsibility of one or another of the co-authors of the paper. The references of the specimens are the ones of the archaeological contexts from which they come from, as indicated in Table 1. Quanhucun et Wuzhuangguoliang specimens are deposited in the Shaanxi Provincial Institute of Archaeology, 31# Leyou Road, Xi’an 710054, China, under the responsability of one of the co-authors, Pr Hu Songmei. Xianwanggang specimens are deposited in the Institute of Archaeology, Chinese Academy of Social Science, 27 Wangfujing Street, Beijing, 20 100710, Beijing, China, under the responsibility of one of the co-authors, Pr Yuan Jing.

### Data acquisition

A total of 96 modern (S1 Table) mandibles, together with 5 Chinese and 5 Cypriot archaeological mandibles were analyzed using two-dimensional landmark based geometric morphometric approaches from standardized photographs taken from the vestibular view. With the help of small spirit levels, the mandibles were positioned in such a way that the plane between the lateral edge of first lower molar and the lateral face of the horizontal *ramus* at the level of the fourth/third lower premolar was horizontal; and the plane between the lateral edges of the ventral and anterior ridges of the vertical *ramus* was also horizontal. Coordinates of 11 landmarks were recorded (S5 Fig, S5 Table) using TpsDig 2 [35]. Data from this study are available at Labarchives (doi: 10.6070/H4NK3C1B). Coordinates were superimposed using a generalized procrustes analysis [36,37] and size (centroid size) and shape (coordinates after superimposition) parameters were analysed separately.

### Statistical analyses

Differences in shape and size between the modern taxa were respectively tested using MANOVA and Kruskall-Wallis test. Shape proximities between the groups were visualised using a neighbour joining network computed from the Mahalanobis D^2^ distances and differences in size variation between taxa were visualised using boxplots. Identifications of the nine archaeological mandibles (five from China and four from Cyprus) were made using predictive linear discriminant analyses (LDA) using *Felis silvestris* (including both subspecies) and *P*. *bengalensis* as the two alternatives. Results of discriminant analyses are known to be affected by the number of specimens by group. To limit the potential bias induced by our unbalanced dataset that included more *F*. *silvestris* than *P*. *bengalensis*, all the identifications were based on 100 random selections of specimens of *F*. *silvestris* to match the number of specimens of *P*. *bengalensis*, and only the discriminant analyses with the higher leave-one out cross validation percentages were retained (above the third quartile of the distribution). Results of the discriminant analyses are described in S3 Table, with the probability of being identified as *P*. *bengalensis* corresponding to the percentage of time where the specimen was identify to this species during the re-sampling process. Before any shape analyses (MANOVA and LDA) were undertaken, a dimensionality reduction was applied [38] by selecting the N first components maximising the discrimination between groups. Morphometric and statistical analyses were performed in R v 2.15.2 (R development Core Team) and the ‘Rmorph’ [39] and ‘ape’ [40] libraries.

## Supporting Information

S1 Fig Drawing of the «ash pit» H3 at Wuzhuangguoliang (Jinghian county, Shaanxi prov.), with the animal deposits comprising 5 hare skeletons, a weasel mandible and the almost complete cat skeleton, and, below, enlargement on the cat skeleton dated to 5267–4871 cal BP (Table 1) (redrawn after the drawings and photos of Hu and Sun, 2005). (PDF)

S2 Fig Photographs of the skull of the small felid found in the H3 refuse pit at Wuzhuangguoliang archaeological site (Jingbian county, Shaanxi prov.): ventral (a), left lateral (b), dorsal (c) and right lateral (d) views. Pictures J-D Vigne.
(PDF)

S3 Fig Results of the Linear discriminant analysis between *P*. *bengalensis* and *F*. *silvestris*. Distribution of the specimens, visualization of the mandible shape between the two species along the discriminant axis, and lateral views of mandibles of the two species (photo A. E.).
(PDF)

S4 Fig Boxplot of centroid size for the five mandibles of PPN small felid cats from Shillourokambos, Cyprus (A), compared with modern domestic cats (Dom), leopard cats (*Pb*), wildcats (*Fs*), and SW Asian cats (*Fsl*). (PDF)

S5 Fig Location of the 11 landmarks used in the geometric morphometric analyses of the cat mandibles for this study. J.-D. Vigne, A. Evin, N. Soulages. A formal description of the landmarks can be found in S5 Table.
(PDF)

S1 Table List of the modern specimens used as reference for this research. J.-D. Vigne, A. Evin, N. Soulage. Abbreviations: F, female; IVPP, Institute of Vertebrate Paleontology and Paleonanthropology, Chinese Academy of Sciences, Beijing; IZCAS, Institute of Zoology, Chinese Academy of Sciences, Beijing; M, male; MNHN, Muséum national d'Histoire Naturelles, collections d'anatomie comparée, Paris.
(PDF)

S2 Table Measurements of the complete skeleton of a small felid found in the H3 refuse pit at Wuzhuangguoliang (Jingbian county, Shaanxi prov.), and dated to 5267–4871 cal BP. Measurements are in millimetres (or millilitres for the volume of the brain case) and they have been recorded according to von den Driesch (1976, *A guide to the measurement of animal bones from archaeological sites*. Harvard: Peabody Museum of Archaeology and Ethnology, 136 p). Skull measurements are the mean of five values. All measurements were taken by J.-D. Vigne.
(PDF)

S3 Table Result of the identification of the nine Chinese and Cyprus archaeological specimens. (PDF)

S4 Table Inventory of small felids excavated from Early Neolithic to Early Bronze Age sites in China. Yu Chong, Yuan Jing, K. Dobney, J.-D. Vigne (unpubl. Database).
(PDF)

S5 Table List and description of the 11 landmarks used in this paper for the geometric morphometric analyses of the cat mandibles. A visual representation of the landmarks can be found in S5 Fig. J.-D. Vigne & A. Evin
(PDF)

S1 Text Permission from the Redlist UICN for the open-access journal PLOS ONE to publish the map of Fig 1 under the Creative Commons Attribution License (CCAL) CC BY 4.0. (PDF)
