# Supplementary material for: Earliest “Domestic” Cats in China Identified as Leopard Cat (Prionailurus bengalensis)
Source: PLoS One. 2016 Jan 22;11(1):e0147295. doi: 10.1371/journal.pone.0147295 (PMC4723238; doi:10.1371/journal.pone.0147295)

S1 Fig: Drawing of the «ash pit» H3 at Wuzhuangguoliang (Jinghian county, Shaanxi prov.), with the animal deposits comprising 5 hare skeletons, a weasel mandible and the almost complete cat skeleton, and, below, enlargement on the cat skeleton dated to 5267-4871 cal BP (Table 1) (redrawn after the drawing and photos of Hu and Sun, 2005).

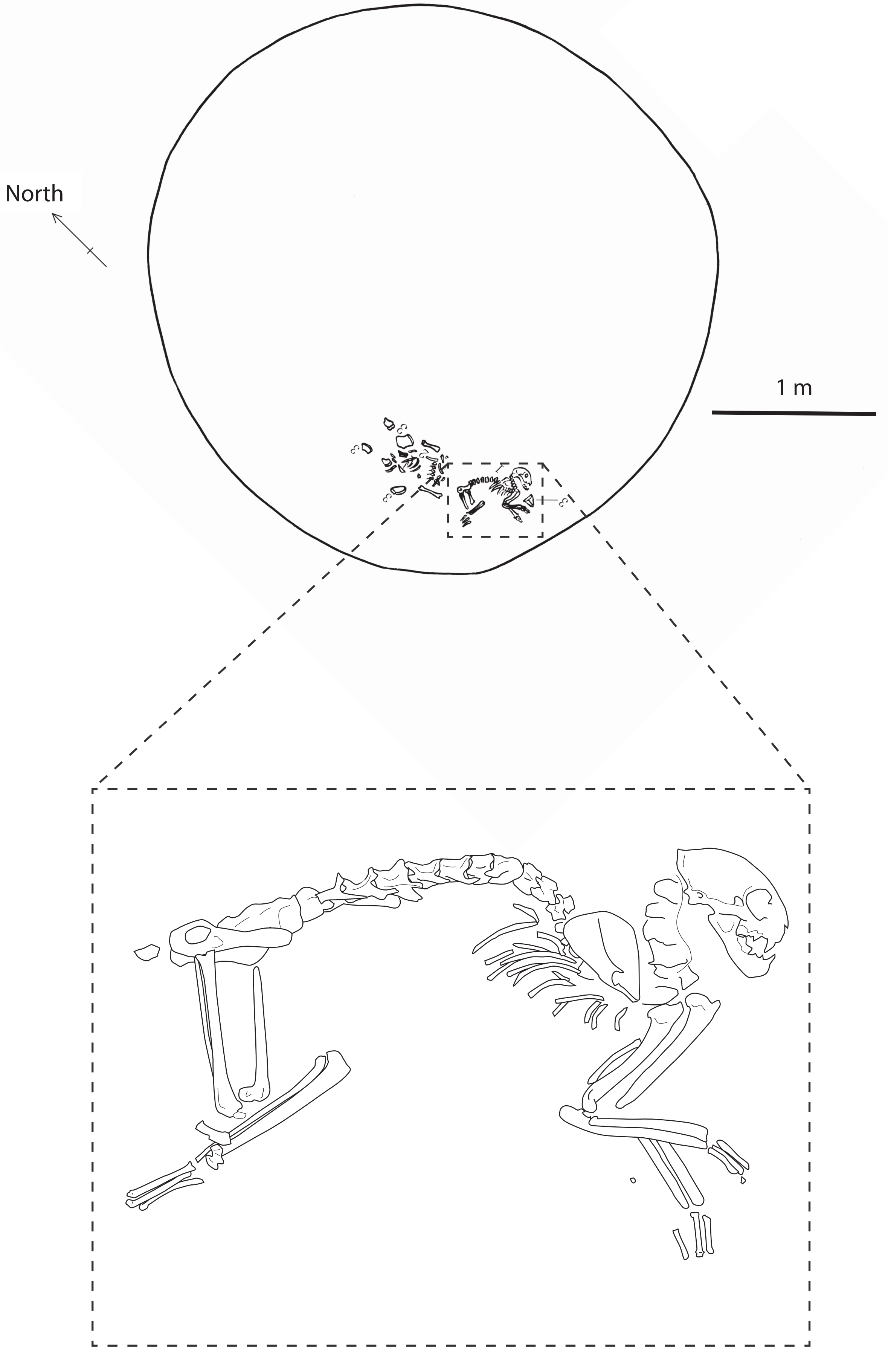

Supplement: S1 Fig — (PDF) [file pone.0147295.s001.pdf]
