# Supplementary figures and images for: Earliest “Domestic” Cats in China Identified as Leopard Cat (Prionailurus bengalensis)
Source: PLoS One. 2016 Jan 22;11(1):e0147295. doi: 10.1371/journal.pone.0147295 (PMC4723238; doi:10.1371/journal.pone.0147295)

S2 Fig  
Vigne et al

Skull of the small  
felid of the  
Wuzhuangguoliang  
site

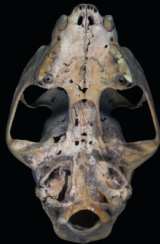

a

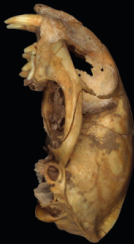

b

1 cm

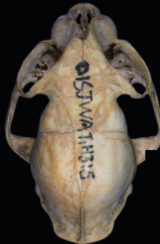

c

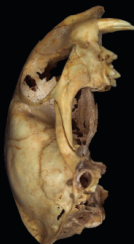

d

Supplement: S2 Fig — Pictures J-D Vigne. (PDF) [file pone.0147295.s002.pdf]
