# Supplementary material for: Earliest “Domestic” Cats in China Identified as Leopard Cat (Prionailurus bengalensis)
Source: PLoS One. 2016 Jan 22;11(1):e0147295. doi: 10.1371/journal.pone.0147295 (PMC4723238; doi:10.1371/journal.pone.0147295)
Supplement: S3 Fig — Distribution of the specimens, visualization of the mandible shape between the two species along the discriminant axis, and lateral views of mandibles of the two species (photo A. E.). (PDF) [file pone.0147295.s003.pdf]

S3 Fig (Vigne et al.)

Results of the Linear discriminant analysis between *P. bengalensis* and F. *silvestris*. Distribution of the specimens, visualization of the mandible shape between the two species along the discriminant axis, and lateral views of mandibles of the two species (photo A. E.)

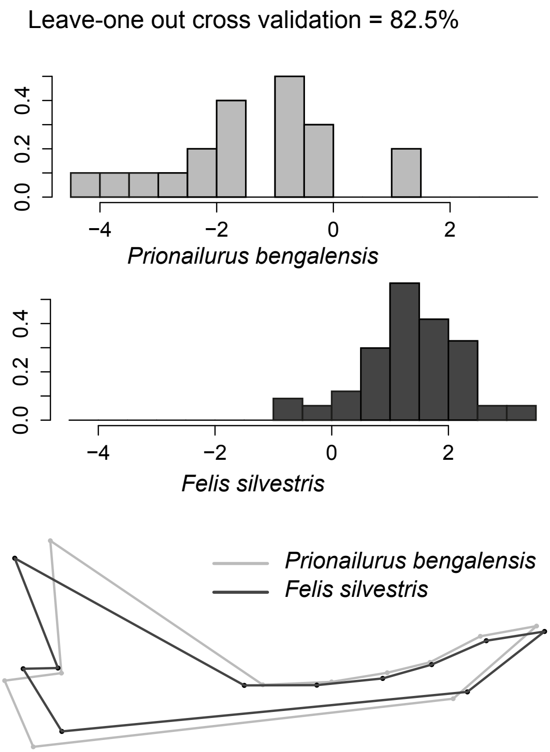


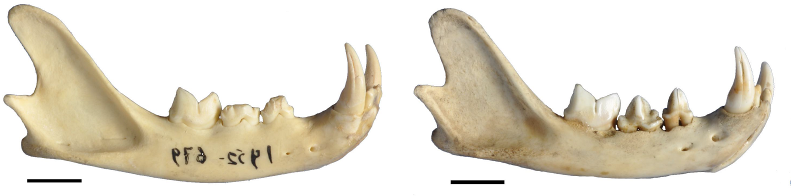

*Felis silvestris*
MNHN 1952-679,
Beni Abbes, Algeria

*Prionailurus bengalensis*
MNHN 1893-153,
Sichuan, China
