# Supplementary material for: Earliest “Domestic” Cats in China Identified as Leopard Cat (Prionailurus bengalensis)
Source: PLoS One. 2016 Jan 22;11(1):e0147295. doi: 10.1371/journal.pone.0147295 (PMC4723238; doi:10.1371/journal.pone.0147295)

S4 Fig (Vigne et al)

Boxplot of centroid size for the five mandibles of PPN small felid cats from Shillourokambos, Cyprus (A), compared with modern domestic cats (Dom), leopard cats (Pb), wildcats (Fs), and SW Asian cats (Fsl).

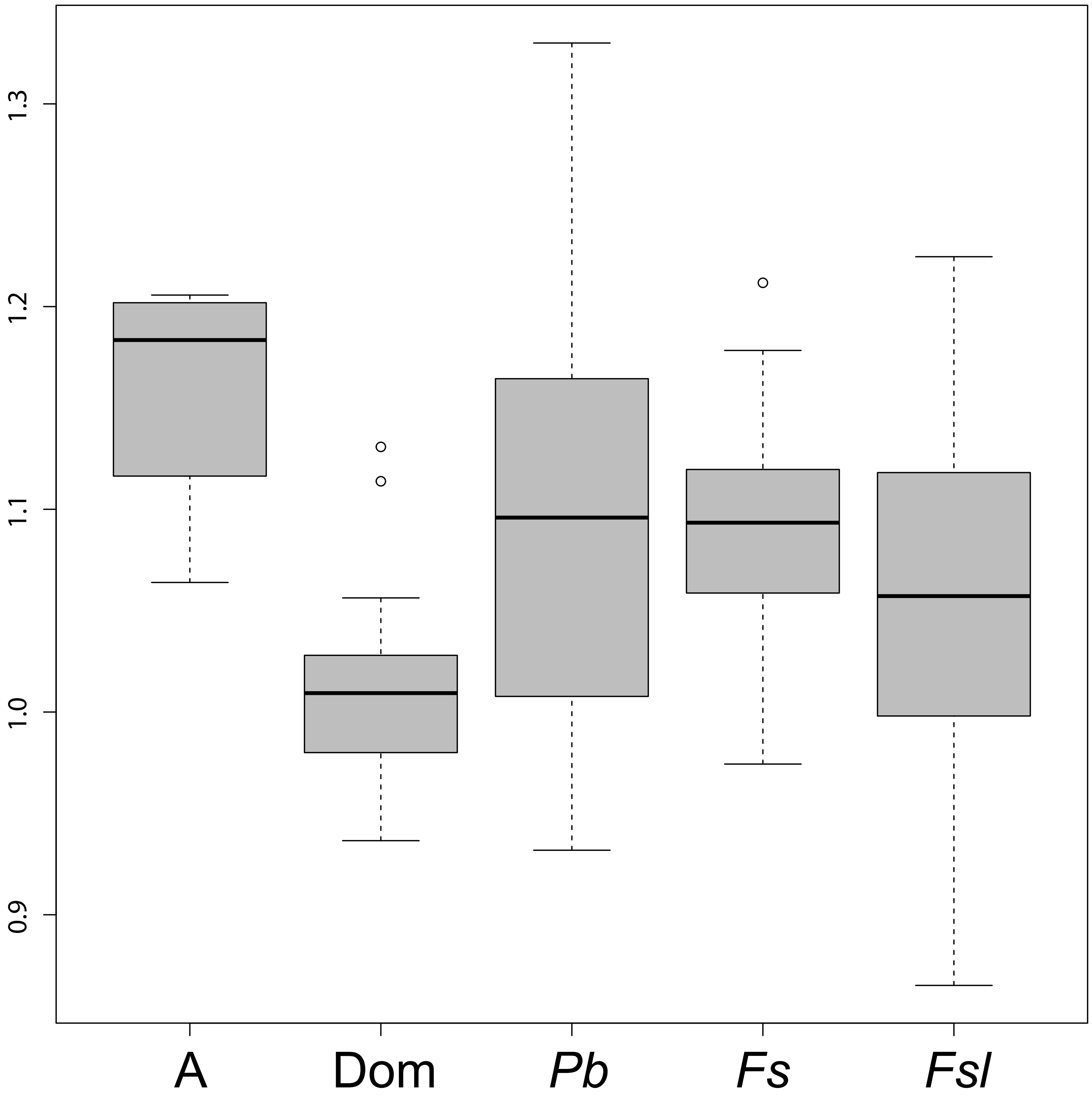

Supplement: S4 Fig — (PDF) [file pone.0147295.s004.pdf]
