# Supplementary material for: Earliest “Domestic” Cats in China Identified as Leopard Cat (Prionailurus bengalensis)
Source: PLoS One. 2016 Jan 22;11(1):e0147295. doi: 10.1371/journal.pone.0147295 (PMC4723238; doi:10.1371/journal.pone.0147295)

S5 Fig (Vigne et al.)

Location of the 11 landmarks used in the geometric morphometric analyses of the cat mandibles for this study. J.-D. Vigne, A. Evin, N. Soulages. A formal description of the landmarks can be found in S5 Table.

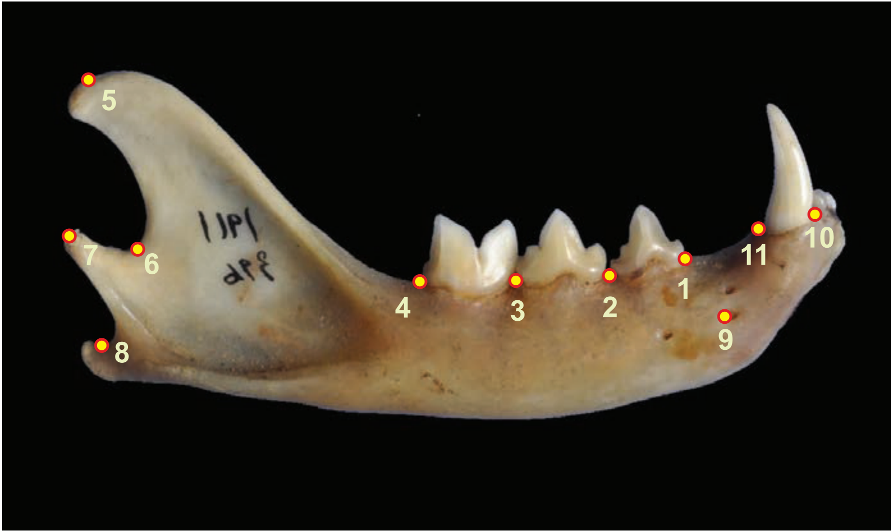

Supplement: S5 Fig — J.-D. Vigne, A. Evin, N. Soulages. A formal description of the landmarks can be found in S5 Table. (PDF) [file pone.0147295.s005.pdf]
