# Supplementary material for: Earliest “Domestic” Cats in China Identified as Leopard Cat (Prionailurus bengalensis)
Source: PLoS One. 2016 Jan 22;11(1):e0147295. doi: 10.1371/journal.pone.0147295 (PMC4723238; doi:10.1371/journal.pone.0147295)
Supplement: S1 Table — J.-D. Vigne, A. Evin, N. Soulage. Abbreviations: F, female; IVPP, Institute of Vertebrate Paleontology and Paleonanthropology, Chinese Academy of Sciences, Beijing; IZCAS, Institute of Zoology, Chinese Academy of Sciences, Beijing; M, male; MNHN, Muséum national d'Histoire Naturelles, collections d'anatomie comparée, Paris. (PDF) [file pone.0147295.s006.pdf]

S1 Table (Vigne et al.)

List of the modern specimens used as reference for this research. J.-D. Vigne, A. Evin, N. Soulage. Abbreviations: F, female; IVPP, Institute of Vertebrate Paleontology and Paleonanthropology, Chinese Academy of Sciences, Beijing; IZCAS, Institute of Zoology, Chinese Academy of Sciences, Beijing; M, male; MNHN, Muséum national d'Histoire Naturelles, collections d'anatomie comparée, Paris.

#	Institution	Code	Sex	Genus	Species	Subspecies	Country
1	MNHN	MNHN-1874-371	?	*Felis*	*catus*		
2	MNHN	MNHN-1884-1409	?	*Felis*	*catus*		
3	MNHN	MNHN-1901-107	?	*Felis*	*catus*		
4	MNHN	MNHN-1902-1032	M	*Felis*	*catus*		
5	MNHN	MNHN-1902-1033	F	*Felis*	*catus*		
6	MNHN	MNHN-1902-805	?	*Felis*	*catus*		
7	MNHN	MNHN-1909-381	F	*Felis*	*catus*		
8	MNHN	MNHN-1923-2293	?	*Felis*	*catus*		
9	MNHN	MNHN-1987-21	?	*Felis*	*catus*		
10	MNHN	MNHN-1987-22	?	*Felis*	*catus*		
11	MNHN	MNHN-A-1756	?	*Felis*	*catus*		
12	MNHN	MNHN-A-1773	?	*Felis*	*catus*		
13	MNHN	MNHN-A3417	?	*Felis*	*catus*		
14	MNHN	MNHN-1874-494	M	*Prionailurus*	*bengalensis*		China
15	MNHN	MNHN-1891-396	?	*Prionailurus*	*bengalensis*		Chinese Tibet
16	MNHN	MNHN-1893-153	?	*Prionailurus*	*bengalensis*		China
17	MNHN	MNHN-1893-154	?	*Prionailurus*	*bengalensis*		China
18	MNHN	MNHN-1895-523	?	*Prionailurus*	*bengalensis*		China
19	MNHN	MNHN-1954-293	F	*Prionailurus*	*bengalensis*		Asia
20	MNHN	MNHN-1955-28	F	*Prionailurus*	*bengalensis*		Asia
21	MNHN	MNHN-1962-1674	F	*Prionailurus*	*bengalensis*		?
22	MNHN	MNHN-1967-1696	?	*Prionailurus*	*bengalensis*		Asia
23	MNHN	MNHN-1995-1243	?	*Prionailurus*	*bengalensis*		Mongolia
24	IVPP	IVPP_100		*Prionailurus*	*bengalensis*		
25	IVPP	IVPP_50		*Prionailurus*	*bengalensis*		
26	IVPP	IVPP_51		*Prionailurus*	*bengalensis*		
27	IVPP	IVPP_61		*Prionailurus*	*bengalensis*		
28	IZCAS	IZCAS_0550		*Prionailurus*	*bengalensis*		
29	IZCAS	IZCAS_1032		*Prionailurus*	*bengalensis*		
30	IZCAS	IZCAS_1032		*Prionailurus*	*bengalensis*		
31	IZCAS	IZCAS_2593		*Prionailurus*	*bengalensis*		
32	IZCAS	IZCAS_no_re		*Prionailurus*	*bengalensis*		
33	IZCAS	IZCAS_Ou-ho		*Prionailurus*	*bengalensis*		
34	MNHN	MNHN-1911-396	?	*Felis*	*silvestris*	*silvestris*	France
35	MNHN	MNHN-1911-397	?	*Felis*	*silvestris*	*silvestris*	France
36	MNHN	MNHN-1934-1417	M	*Felis*	*silvestris*	*silvestris*	France
37	MNHN	MNHN-1950-861	M	*Felis*	*silvestris*	*silvestris*	France
38	MNHN	MNHN-1952-537	M	*Felis*	*silvestris*	*silvestris*	France
39	MNHN	MNHN-1955-140	F	*Felis*	*silvestris*	*silvestris*	France
40	MNHN	MNHN-1955-141	M	*Felis*	*silvestris*	*silvestris*	France
41	MNHN	MNHN-1962-4160	M	*Felis*	*silvestris*	*silvestris*	France
42	MNHN	MNHN-1971-55	M	*Felis*	*silvestris*	*silvestris*	France
43	MNHN	MNHN-1972-617	?	*Felis*	*silvestris*	*silvestris*	France
44	MNHN	MNHN-1983-798	M	*Felis*	*silvestris*	*silvestris*	France
45	MNHN	MNHN-1987-122	?	*Felis*	*silvestris*	*silvestris*	France

46	MNHN	MNHN-1987-272	?	*Felis*	*silvestris*	*silvestris*	France
47	MNHN	MNHN-1990-470	?	*Felis*	*silvestris*	*silvestris*	France
48	MNHN	MNHN-1990-86	?	*Felis*	*silvestris*	*silvestris*	France
49	MNHN	MNHN-1991-703	F	*Felis*	*silvestris*	*silvestris*	France
50	MNHN	MNHN-1992-1642	F	*Felis*	*silvestris*	*silvestris*	France
51	MNHN	MNHN-1992-1643	M	*Felis*	*silvestris*	*silvestris*	France
52	MNHN	MNHN-1992-1644	F	*Felis*	*silvestris*	*silvestris*	France
53	MNHN	MNHN-1992-2015	M	*Felis*	*silvestris*	*silvestris*	France
54	MNHN	MNHN-1992-2016	F	*Felis*	*silvestris*	*silvestris*	France
55	MNHN	MNHN-1992-2018	M	*Felis*	*silvestris*	*silvestris*	France
56	MNHN	MNHN-1992-2019	M	*Felis*	*silvestris*	*silvestris*	France
57	MNHN	MNHN-1992-2021	F	*Felis*	*silvestris*	*silvestris*	France
58	MNHN	MNHN-1993-1664	?	*Felis*	*silvestris*	*silvestris*	France
59	MNHN	MNHN-1994-807	F	*Felis*	*silvestris*	*silvestris*	France
60	MNHN	MNHN-2006-240	F	*Felis*	*silvestris*	*silvestris*	France
61	MNHN	MNHN-2006-238	?	*Felis*	*silvestris*	*silvestris*	France
62	MNHN	MNHN-2010-630	F	*Felis*	*silvestris*	*silvestris*	France
63	MNHN	MNHN-1932-103	F	*Felis*	*silvestris*	*lybica*	Marocco
64	MNHN	MNHN-1932-104	M	*Felis*	*silvestris*	*lybica*	Marocco
65	MNHN	MNHN-1932-105	M	*Felis*	*silvestris*	*lybica*	Marocco
66	MNHN	MNHN-1933-2321	F	*Felis*	*silvestris*	*lybica*	Ethiopia
67	MNHN	MNHN-1934-938	?	*Felis*	*silvestris*	*lybica*	Tchad
68	MNHN	MNHN-1950-252	M	*Felis*	*silvestris*	*lybica*	Togo
69	MNHN	MNHN-1950-253	M	*Felis*	*silvestris*	*lybica*	Togo
70	MNHN	MNHN-1950-254	M	*Felis*	*silvestris*	*lybica*	Togo
71	MNHN	MNHN-1950-256	M	*Felis*	*silvestris*	*lybica*	Togo
72	MNHN	MNHN-1950-257	F	*Felis*	*silvestris*	*lybica*	Togo
73	MNHN	MNHN-1950-258	F	*Felis*	*silvestris*	*lybica*	Togo
74	MNHN	MNHN-1950-259	M	*Felis*	*silvestris*	*lybica*	Togo
75	MNHN	MNHN-1950-260	F	*Felis*	*silvestris*	*lybica*	Togo
76	MNHN	MNHN-1950-262	F	*Felis*	*silvestris*	*lybica*	Togo
77	MNHN	MNHN-1950-55	M	*Felis*	*silvestris*	*lybica*	Togo
78	MNHN	MNHN-1951-1019	?	*Felis*	*silvestris*	*lybica*	Tchad
79	MNHN	MNHN-1952-679	?	*Felis*	*silvestris*	*lybica*	Algeria
80	MNHN	MNHN-1953-392	M	*Felis*	*silvestris*	*lybica*	Algeria
81	MNHN	MNHN-1959-1806	?	*Felis*	*silvestris*	*lybica*	Tchad
82	MNHN	MNHN-1961-620	?	*Felis*	*silvestris*	*lybica*	?
83	MNHN	MNHN-1969-459	M	*Felis*	*silvestris*	*lybica*	Ethiopia
84	MNHN	MNHN-1969-460	F	*Felis*	*silvestris*	*lybica*	Ethiopia
85	MNHN	MNHN-1970-289	M	*Felis*	*silvestris*	*lybica*	Senegal
86	MNHN	MNHN-1972-401	M	*Felis*	*silvestris*	*lybica*	Ethiopia
87	MNHN	MNHN-1972-402	M	*Felis*	*silvestris*	*lybica*	Ethiopia
88	MNHN	MNHN-1972-403	F	*Felis*	*silvestris*	*lybica*	Ethiopia
89	MNHN	MNHN-1973-137	M	*Felis*	*silvestris*	*lybica*	Ethiopia
90	MNHN	MNHN-1973-138	M	*Felis*	*silvestris*	*lybica*	Ethiopia
91	MNHN	MNHN-1973-139	M	*Felis*	*silvestris*	*lybica*	Ethiopia

92	MNHN	MNHN-1973-140	M	*Felis*	*silvestris*	*lybica*	Ethiopia
93	MNHN	MNHN-1973-141	F	*Felis*	*silvestris*	*lybica*	Ethiopia
94	MNHN	MNHN-1974-326	?	*Felis*	*silvestris*	*lybica*	Nigeria
95	MNHN	MNHN-1978-138	M	*Felis*	*silvestris*	*lybica*	Ethiopia
96	MNHN	MNHN-1995-3151	?	*Felis*	*silvestris*	*lybica*	Mauritania
97	MNHN	MNHN-1995-444	F	*Felis*	*silvestris*	*lybica*	Senegal
98	MNHN	MNHN-1995-446	F	*Felis*	*silvestris*	*lybica*	Senegal
99	MNHN	MNHN-1995-448	F	*Felis*	*silvestris*	*lybica*	Senegal
100	MNHN	MNHN-1995-449	F	*Felis*	*silvestris*	*lybica*	Senegal
