# Supplementary material for: Earliest “Domestic” Cats in China Identified as Leopard Cat (Prionailurus bengalensis)
Source: PLoS One. 2016 Jan 22;11(1):e0147295. doi: 10.1371/journal.pone.0147295 (PMC4723238; doi:10.1371/journal.pone.0147295)
Supplement: S2 Table — Measurements are in millimetres (or millilitres for the volume of the brain case) and they have been recorded according to von den Driesch (1976, A guide to the measurement of animal bones from archaeological sites. Harvard: Peabody Museum of Archaeology and Ethnology, 136 p). Skull measurements are the mean of five values. All measurements were taken by J.-D. Vigne. (PDF) [file pone.0147295.s007.pdf]

S2 Table (Vigne et al)

Measurements of the complete skeleton of a small felid found in the H3 refuse pit at Wuzhuangguoliang (Jingbian county, Shaanxi prov.),and dated to 5267-4871 cal BP.

Skull										
1	2	3	4	5	6	7	8	9	10	11a
100.0	89.0	83.1	28.3	52.6	79.5	48.4	66.4	33.3	24.1	39.2
12	13	14	BP4/	16	17	18	19	20	21	22
21.6	20.4	10.6	4.7	21.4	12.6	38.9	22.5	14.4	12.0	42.0
23	25	26	27	28	29	31	32	33		
63.4	15.8	37.5	22.38	26.53	26.69	27.23	29.31	31±1.4		

Mandib.	1	2	3	4	5	BM/1	7	8	9	10
left	61.23	59.39	54.32	52.91	19.7	3.8	8.1	29.16	10.71	10.33
right	61.8	60.36	54.06	54.41	20.3	3.8	7.9	28.8	10.78	9.48

Atlas	
GL	GB
18.9	36.44

Axis					
LCDe	LAPa	H	BFcr	SBV	Bfacd
25.24	27.33	20.68	15.93	13.07	17.01

Scapula	HS	GLP	BG	SLC
left		14.6	9.36	12.89
right	74.5	14.3	10.13	12.78

Humerus	GL	GLC	SD	Bd	Td
left	107.1	106.8	7.66	18.3	10.61
right	107.5	106.4	7.65	18.2	10.24

Ulna	LO	SDO	DPA	Bp
left	12.5	9.42	11.6	8.65
right	12.44	9.28	11.63	8.5

Radius	GL	Bp	Tp	KD
left	94.52	8.35	6.1	5.85
right	94.47	8.36	6.09	5.76

Mcarpal	GL	Bp	B	Bd
II left	27.14	4.19	2.48	4.82
II right	27.05	4.5	2.65	4.54
III left	33.25	4.68	3.0	4.9
III right	32.7	4.43	2.85	4.5
IV right	35.9	5.78	3.36	5.1
V right	30.95	4.37	2.9	5.1

Pelvis	GL	SB	SH	LA	LfO	LS
left	87.5	5.38	11.7	11.3	20.03	28.44
right	87.4	5.2	11.6	11.25	19.98	26.6

Femur	GL	GLC	Bp	SD	Bd	Td
left	119.8	119.8	20.53	8.98	18.27	18.32
right	119.4	119.9	20.42	9.96	18.34	18.5

Tibia	GL	Ll	Bp	Dp	SD	Bd	Td
left	122.7	119.6		19.23	7.94	14.19	9.75
right		118.4	19.34	19.25	7.96		

Calca.	GL	GB
left	28.06	12.78
right	27.6	12.65

Mtarsal	GL	Bp	B	Bd
III left	52.68	7.04	4.58	5.97
III right	53.2		4.45	5.67
IV left	53.01			5.36
IV right	53.07			
V left	50.2			
V right	51.05			
