# Supplementary material for: Earliest “Domestic” Cats in China Identified as Leopard Cat (Prionailurus bengalensis)
Source: PLoS One. 2016 Jan 22;11(1):e0147295. doi: 10.1371/journal.pone.0147295 (PMC4723238; doi:10.1371/journal.pone.0147295)
Supplement: S3 Table — (PDF) [file pone.0147295.s008.pdf]

S3 Table (Vigne et al)
Result of the identification of the nine Chinese and Cyprus archaeological specimens.

**Archaeological specimens**	**Origin**	**Number of LDA retained**	**Limit to concider a LDA**	**% of identifications to *P. bengalensis***	**min Proba**	**max Proba**	**Identification**
Quanhucun, H172_A	China	42	75%	100%	81%	99%	*P. bengalensis*
Quanhucun, H172_B	China	25	68.13%	100%	53%	91%	*P. bengalensis*
Wuzhuangguoliang, H3	China	32	77.5%	100%	75%	99%	*P. bengalensis*
Xiawanggang, H134	China	37	75%	100%	98%	100%	*P. bengalensis*
Xiawanggang, Layer 7	China	34	80%	100%	56%	100%	*P. bengalensis*
Felis_S3_St238_2002	Cyprus	31	77.5%	100%	94%	100%	*F. silvestris*
Felis_S3_St238_3	Cyprus	31	77.5%	100%	98%	100%	*F. silvestris*
Felis_St283_dt	Cyprus	31	77.5%	100%	100%	100%	*F. silvestris*
Felis_St283_gch	Cyprus	31	77.5%	94%	52%	98%	*F. silvestris*
