# Supplementary material for: Earliest “Domestic” Cats in China Identified as Leopard Cat (Prionailurus bengalensis)
Source: PLoS One. 2016 Jan 22;11(1):e0147295. doi: 10.1371/journal.pone.0147295 (PMC4723238; doi:10.1371/journal.pone.0147295)
Supplement: S4 Table — Yu Chong, Yuan Jing, K. Dobney, J.-D. Vigne (unpubl. Database). (PDF) [file pone.0147295.s009.pdf]

Site (Chinese / English)		Province	County	Date Category	Taxon (Chinese/English)		NISP	MNI	Reference
济南长清月庄	Changqingyuezhuang	Shandong	Jinan		猫科	Feildae	4	1	Song,Y..2007. Analysis of the Animal Remains from the site Changqingyuezhuang excavated from 2003, Jinan.In:School of Archaeology and Museology,Penking University(Eds.). A Collection of Studies on Archaeology VII.Science Press.Beijing.pp.519-531.
舞阳贾湖	Jiahu	Henan	Wuyang	Early Neolithic	豹猫	*Prionailurus bengalensis*			Huang,W..1999.Faunal Assemblge.In:Henan Provincial Institute of Cultural Relics and Archaeology(Eds.).Wuyang Jiahu.Science Press.Beijing.pp.785-805.
临潼白家村	Baijiacun	Shaanxi	Lintong		猫	Feildae	4		周本雄：《白家村遗址动物遗骸鉴定报告》，见中国社会科学院考古研究所编著：《临潼白家村》，第123～126页，成都：巴蜀书社，1994年。
白城双塔	Shuangta	Jilin	Baicheng		猫科	Feildae		1	Zhang，M..2011.：The Pattern of Animal Exploitation during the Period I of Shuangta Site, Northeastern China.Jilin University Unpublished 吉林大学硕士学位论文。
农安左家山	Zuojiashan	Jilin	Nong'an		草原野猫	*Felis silvestris*	6	2	Chen,Q.1993.The Identification of Animal Bones Found in Zuojiashan Site of Nongan County and its Trace Study.In:The Department of Archaeology, Jilin University(Eds).Collection of Green Fruits.Knowledge
凉城石虎山Ⅰ	Shihushan I	Inner Mongolia	Liangcheng		豹猫	*Prionailurus bengalensis*	3	1	Huang,Y..2001.《石虎山I遗址动物骨骼鉴定与研究》，Inner Mongolia Autonomous Institute of Cultural Relics and Archaeology,Japan Kyoto Society of Chinese Archaeology(Eds).Archaeological Excavations at Daihai II, 第489～513页，北京：科学出版社.
潍坊前埠下	Qianbuxia	Shandong	Weifang		猫	Feildae			Kong,Q..2000.Animal Remains from Neolithic Site Qianpuxia.In:Shandong Provincial Institute of Cultural Relics and Archaeology(Eds.).Archaeology Report on the Shandong Highway 1997.Science
兖州王因	Wangyin	Shandong	Yanzhou		野猫	*Felis silvestris*			Zhou,B..2000.Animal Remains from Neolithihc Site Wangyin in Yanzhou County, Shandong.In:The Institute of Archaeology Chinese Academy of Social Sciiences(Eds.).Excavation of the Wangyin Site in
濮阳西水坡	Xishuipo	Henan	Puyang		猫科动物	Feildae	9	4	Lv,P.,Yuan,J.,Yang,M..2012.Identification and Study of the Animal Remains from Xishuipo Site.In:Cultural Relic and Archaeology Research Institute of Henan Province,Cultural Relic Preservation Adminisration of Puyang City(Eds).Xishuipo in Puyang.Cultural Relics Press.Beijing.pp.659-696.
淅川下王冈	Xiawanggang	Henan	Xichuan	Middle Neolithic	豹猫	*Prionailurus bengalensis*			贾兰坡、张振标：《河南淅川下王冈遗址中的动物群》，见The Henan Provincial Institute of Archaeology,The Henan Group of The Archaeological Team ,The Office for Programme of the Works in the Changjiang River Valley:Xiawanggang Site at Xichuan, 第429～439页，北京：文物出版社，1989年。
西安半坡	Banpo	Shaanxi	Xi'an		狸	*Felis* sp.	1		Li, Y.,Han,D.,1959.Animal bones from the Neolithic site of Banpo in Xi'an, Shaanxi. Gu jizhui dongwu yu gurenlei[Paleoverebrata et Palelanthropologia]1 (4), 173–185.
临潼姜寨	Jiangzhai	Shaanxi	Lintong		猫	Feildae	1	1	Qi,G.1988.Analysis of the faunal assemblage from the Neolithic site of Jiangzhai.In:Xi'an Banpo Museum, Shaanxi Institute of Archaeology, Lintong County Museum（Eds.）.Jiangzhai.Cultural Reclis
临潼姜寨	Jiangzhai	Shaanxi	Lintong		猫	Feildae	1	1	Qi,G.1988.Analysis of the faunal assemblage from the Neolithic site of Jiangzhai.In:Xi'an Banpo Museum, Shaanxi Institute of Archaeology, Lintong County Museum（Eds.）.Jiangzhai.Cultural Reclis
靖边五庄果墚	Wuzhuangguoliang	Shaanxi	Jingbian		草原斑猫	*Felis silvestris*		1	Hu,S.,Sun,Z..2005.The Faunal Remains of the Wuzhuangguoliang Site and Its Palaeoenvirment Analysis, 《考古与文物》2005年第6期, 第72～84页。
高陵东营	Dongying	Shaanxi	Gaoling		猫	Feildae	1	1	Hu,S.,2010.Analysis on the Animal Remains from Dongying Site,Gaoying.In:Shaanxi Provincial Institute of Archaeology and Research Center for Cultural Heritage and Archaeology Study of Northwest University(Eds.).Dongying in Gaoling.Science Press.Beijing.pp.147-200.
秦安大地湾	Dadiwan	Gansu	Qin'an		豹猫	*Prionailurus bengalensis*	1		Qi,G.,Lin,Z.,An,J..2006.Report on the Identification of Animal Remains from Dadiwan Site.In:Gansu Provincial Institute of Cultural Relics and Archaeology(Eds.).Dadiwan in Qin'an.Cultural Relics Publishing
秦安大地湾	Dadiwan	Gansu	Qin'an		豹猫	*Prionailurus bengalensis*	1		Qi,G.,Lin,Z.,An,J..2006.Report on the Identification of Animal Remains from Dadiwan Site.In:Gansu Provincial Institute of Cultural Relics and Archaeology(Eds.).Dadiwan in Qin'an.Cultural Relics Publishing House.Beijing.pp.861-910.
大连郭家村	Guojiacun	Liaoning	Dalian		野猫	*Felis silvestris*	1		Fu,R..1984.《Study of the Animal Bones Unearthed at Guojiacun》, 《考古学报》第3期, 第331～334页。
泰安大汶口	Dawenkou	Shandong	Tai'an		狸	Felis sp.			李有恒：《大汶口墓群的兽骨及其他动物骨骼》，见山东省文物管理处、济南市博物馆编：《大汶口》，第156～158页，北京：文物出版社，1974年。
潍县鲁家口	Lujiakou	Shandong	Weixian		猫	Felis catus			Zhou,B..1985.Animal Remains from Lujiakou Site at Weixian County,Shandong.Kaogu Xuebao[Acta
兖州西吴寺	Xiwusi	Shandong	Yanzhou		豹猫	*Prionailurus bengalensis*	1		Lu,H..1990.Report on the Identification of Animal Bones from Xiwusi Site.In:The Training Class of Leading Archaeological Personnels, the State Buresu of Archaeological Affairs and Museums(Eds.).Xiwusi Site of Yanzhou County.Cultural Relics Publishing House.Beijing.pp.248-249.
兖州六里井	Liulijing	Shandong	Yanzhou		豹猫	*Prionailurus bengalensis*		2	Fan,C..1999.Identification of the animal remains from Liulijing Site.In: The training class of leading archaeological personnels, the State Administration of Cultural Heritage(Eds.).Liulijing Site of Yanzhou
淄博桐林	Tonglin	Shandong	Zibo		猫	Feildae			Huang,Y..2010.A Quantitative Analysis of Faunal Remains and the Development of Animal Domestication.In:Henan Provincial Institute of Cultural Relics and Archaeology(Eds.).Zooarchaeology.Vol
汤阴白营	Baiying	Henan	Tangyin		家猫	*Felis catus*			Zhou,B..1983.Animal Remains from Longshan Cultural Site Baiying in Tangyin County,Henan.In:Editorial Department of Archaeology(Eds.).Papers on Chinese Archaeology (3).Publishing House of Chinese Social
商县紫荆	Zijing	Shaanxi	Shangxian	Late Neolithic	野猫	*Felis silvestris*	1		Wang,Y..1991.The Faunal at Zijing Site and its Paleoenvironmental Significance.In:Zhou,K.(Eds.).Research on Environmental Archaeology.Vol 1.Science Press.Beijing.pp.96-99.
临潼康家	Kangjia	Shaanxi	Lintong		猫	Feildae	1		Liu, L.,Yan, Y.,Qin, X.,2001.Faunal remains from the 1990 excavations at the Longshan site of Kangjia in Lintong, Shaanxi.Huaxia Kaogu[Hua Xia archaeology] (1), 3–24.
丹凤巩家湾	Gongjiawan	Shaanxi	Danfeng		猫科	Feildae	1	1	Hu,S..2001.An Analysis of the animal bone from Gongjiawan Neolithic site, Shaanxi Province.Kaogu yu Wenwu[Archaeology and Cultural Relics](6).pp.53-57.
高陵东营	Dongying	Shaanxi	Gaoling		猫	Feildae	2	1	Hu,S.,2010.Analysis on the Animal Remains from Dongying Site,Gaoying.In:Shaanxi Provincial Institute of Archaeology and Research Center for Cultural Heritage and Archaeology Study of Northwest University(Eds.).Dongying in Gaoling.Science Press.Beijing.pp.147-200.
天水师赵村	Shizhaocun	Gansu	Tianshui		狸	*Felis* sp.			Zhou,B.,1999.Animal Remains from the Sites of Shicunzhao and Xishanping.In:The Institute of Archaeology,Chinese Academy of Social Sciences(Eds), Shizhaocun and Xishanping.The Encyclopeida of China Publishing House.Beijing.pp.335–339.
西宁长宁	Changning	Qinghai	Xining		猞猁	Lynx lynx		7	Li,I..2012.Study on the Animal Remains from the Site of Changning.Jilin University Unpublished Master
西宁长宁	Changning	Qinghai	Xining		猫属	Feildae		1	Li,I..2012.Study on the Animal Remains from the Site of Changning.Jilin University Unpublished Master
偃师二里头	Erlitou	Henan	Yanshi		猫科	Feildae	2	1	Yang,J..2008.Study on the Animal Remains from Erlitou Site.In:The Institute of Archaeology,Chinese Academy of Social Sciences(Eds).Early Bronze Age in China.Science Press.Beijing.pp.470-539.
偃师二里头	Erlitou	Henan	Yanshi		小型猫科	Small Felidae	2	2	Yang,J..2008.Study on the Animal Remains from Erlitou Site.In:The Institute of Archaeology,Chinese Academy of Social Sciences(Eds).Early Bronze Age in China.Science Press.Beijing.pp.470-539.
登封南洼	Nanwa	Henan	Dengfeng		猫	Feildae	2	1	Yu,C..2012.Study and Identification on the Animal Remains of Nanwa Site in Dengfeng.In:Department of Archaeology of Zhengzhou University(Eds.).Nanwa in Dengfeng.Science Press.Beijing.pp.
登封南洼	Nanwa	Henan	Dengfeng	Bronze Age	猫	Feildae	1	1	Yu,C..2012.Study and Identification on the Animal Remains of Nanwa Site in Dengfeng.In:Department of Archaeology of Zhengzhou University(Eds.).Nanwa in Dengfeng.Science Press.Beijing.pp.
登封南洼	Nanwa	Henan	Dengfeng		猫	Feildae	1	1	Yu,C..2012.Study and Identification on the Animal Remains of Nanwa Site in Dengfeng.In:Department of Archaeology of Zhengzhou University(Eds.).Nanwa in Dengfeng.Science Press.Beijing.pp.
榆林火石梁	Huoshiliang	Shanxi	Yulin		豹猫	*Prionailurus bengalensis*	5	2	Hu,S.,Zhang,P.,Yuan,M..2008.A Study on the Faunal Remains from the Huoshiliang Site in Yulin ,Shaanxi.Renleixue Xuebao[Acta Anthropoligica Sinica]Vol 27,No.3.pp.232-248.
