# Supplementary material for: Earliest “Domestic” Cats in China Identified as Leopard Cat (Prionailurus bengalensis)
Source: PLoS One. 2016 Jan 22;11(1):e0147295. doi: 10.1371/journal.pone.0147295 (PMC4723238; doi:10.1371/journal.pone.0147295)
Supplement: S5 Table — A visual representation of the landmarks can be found in S5 Fig. J.-D. Vigne & A. Evin (PDF) [file pone.0147295.s010.pdf]

S5 Table (Vigne et al)
List and description of the 11 landmarks used in this paper for the geometric morphometric analyses of the cat mandibles. A visual representation of the landmarks can be found in S5 Fig. J.-D. Vigne & A. Evin

N°	Description
1	Anterior extremity of the alveolus of P/3
2	Middle point between the alveolus of P/3 and P/4
3	Posterior extremity of the alveolus of P/4
4	Posterior extremity of the alveolus of the M/1
5	Maximum curvature of the coronoid process
6	Deepest point of the curvature of the mandibular incisure
7	Maximum curvature of the articulation
8	Most ventral point of the dorsal incisure of the angular process
9	Most posterior termination of the anterior chin foramen
10	Most anterior point of the alveolus of the canine
11	Most posterior extremity of the alveolus of the canine
