# Supplementary material for: Earliest “Domestic” Cats in China Identified as Leopard Cat (Prionailurus bengalensis)
Source: PLoS One. 2016 Jan 22;11(1):e0147295. doi: 10.1371/journal.pone.0147295 (PMC4723238; doi:10.1371/journal.pone.0147295)
Supplement: S1 Text — (PDF) [file pone.0147295.s011.pdf]

Dear Jean-Denis Vigne,

The IUCN Red List Unit grants permission for the open-access journal PLOS ONE to publish under the Creative Commons Attribution License (CCAL) CC BY 4.0), Figure 1 of the manuscript by Jean-Denis Vigne et al., entitled "Earliest 'domestic' cats in China identified as leopard cat (Prionailurus bengalensis)". This figure has been drawn based on the two followings:

- Felis silvestris (http://dx.doi.org/10.2305/IUCN.UK.2015-2.RLTS.T60354712A50652361.en); map at: http://maps.iucnredlist.org/map.html?id=60354712
- Prionailurus bengalensis (http://dx.doi.org/10.2305/IUCN.UK.2015-4.RLTS.T18146A50661611.en); map at: http://maps.iucnredlist.org/map.html?id=18146

Cambridge, UK, 16th December 2015

All the best,

Catherine

Catherine Sayer
Junior Professional
Red List Unit
IUCN (International Union for Conservation of Nature)
The David Attenborough Building
Pembroke Street
Cambridge CB2 3QZ
United Kingdom
http://iucn.org/
http://www.iucnredlist.org//
